# The DAMASK trial protocol: a pragmatic randomised trial to evaluate whether GPs should have direct access to MRI for patients with suspected internal derangement of the knee

**DOI:** 10.1186/1472-6963-6-133

**Published:** 2006-10-13

**Authors:** Stephen D Brealey, Christine Atwell, Stirling Bryan, Simon Coulton, Helen Cox, Ben Cross, Fiona Fylan, Andrew Garratt, Fiona J Gilbert, Maureen GC Gillan, Maggie Hendry, Kerenza Hood, Helen Houston, David King, Veronica Morton, Jo Orchard, Michael Robling, Ian T Russell, David Torgerson, Valerie Wadsworth, Clare Wilkinson

**Affiliations:** 1 Department of Health Sciences, York Trials Unit, Seebohm Rowntree Building, University of York, Heslington, York YO10 5DD, UK; 2 Department of General Practice, Cardiff University, Neuadd Meirionydd, Heath Park, Cardiff, CF14 4YS, UK; 3 Health Services Management Centre, University of Birmingham, Park House, 40 Edgbaston Park Road, Birmingham, West Midlands, B15 2TT, UK; 4 Department of Health Sciences, Seebohm Rowntree Building, University of York, Heslington, York YO10 5DD, UK; 5 Department of Psychology, Leeds Metropoitan University, Leeds LS1 3HE, UK; 6 Institute of Health Management and Health Economics, University of Oslo, PO Box 1089, Blindern, NO-0317 Oslo, Norway; 7 Department of Radiology, Lilian Sutton Building, University of Aberdeen, Foresterhill, Aberdeen, AB25 2ZD, UK; 8 Cardiff University School of Medicine, North Wales Clinical School, Gwenfro Building, Unit 5, Wrexham Technology Park, Wrexham, LL13 7YP, UK; 9 South East Wales Trials Unit, Centre for Health Sciences Research, Cardiff University, Neuadd Meirionydd, Heath Park, Cardiff, CF14 4YS, UK; 10 X-ray Department, York Hospital, Wigginton Road, York, YO31 8HE, UK; 11 Institute of Medical and Social Care Research, Brigantia Building, Penrallt Road, University of Wales Bangor, Gwynedd, LL57 2AS, UK

## Abstract

**Background:**

Though new technologies like Magnetic Resonance Imaging (MRI) may be accurate, they often diffuse into practice before thorough assessment of their value in diagnosis and management, and of their effects on patient outcome and costs. MRI of the knee is a common investigation despite concern that it is not always appropriate. There is wide variation in general practitioners (GPs) access to, and use of MRI, and in the associated costs. The objective of this study was to resolve uncertainty whether GPs should refer patients with suspected internal derangement of the knee for MRI or to an orthopaedic specialist in secondary care.

**Methods/Design:**

The design consisted of a pragmatic multi-centre randomised trial with two parallel groups and concomitant economic evaluation. Patients presenting in general practice with suspected internal derangement of the knee and for whom their GP was considering referral to an orthopaedic specialist in secondary care were eligible for inclusion. Within practices, GPs or practice nurses randomised eligible and consenting participants to the local radiology department for an MRI examination, or for consultation with an orthopaedic specialist. To ensure that the waiting time from GP consultation to orthopaedic appointment was similar for both trial arms, GPs made a provisional referral to orthopaedics when requesting the MRI examination. Thus we evaluated the more appropriate sequence of events independent of variations in waiting times. Follow up of participants was by postal questionnaires at six, twelve and 24 months after randomisation. This was to ensure that the evaluation covered all events up to and including arthroscopy.

**Discussion:**

The DAMASK trial should make a major contribution to the development of evidence-based partnerships between primary and secondary care professionals and inform the debate when MRI should enter the diagnostic pathway.

## Background

Each year in the United Kingdom (UK) 15% of patients who consult general practitioners (GPs) do so for musculoskeletal disorders. The annual consulting rate for internal derangement of the knee is 32 per 1000 patient years, similar to rheumatoid arthritis [1]. Imaging of the knee is a common musculoskeletal application of Magnetic Resonance Imaging (MRI) [2]. Although there is evidence for the technical [3] and diagnostic [4-6] performance of MRI for knee problems, there is uncertainty about the appropriate use of MRI, in particular when it should enter the diagnostic pathway for patients with suspected internal derangement of the knee [7,8]. This question is crucial to patient diagnosis, management and outcome, and thus to cost-effectiveness.

Systematic reviews have consistently demonstrated that MRI is an accurate diagnostic test for detecting meniscal and cruciate lesions [7-12]. For example, at least 85% of meniscal lesions and 90% of healthy menisci found at arthroscopy are detected with MRI [10]. A trial that included 209 patients with negative MRI results who were randomised for arthroscopic or conservative treatment also concluded that MRI is accurate for the diagnosis of knee injuries [13]. Therefore the evidence supports the use of MRI for diagnosing these common problems. This has led some to suggest that it is valuable to GPs in making appropriate and informed decisions [14,15]. Negative MRI findings could allow GPs to reassure patients, treat them conservatively in primary care, avoid unnecessary orthopaedic referrals and hence reduce waiting times [2,3] and save costs [16]. Alternatively positive MRI findings could confirm GPs' clinical diagnoses and decision to refer to an orthopaedic specialist who would decide whether arthroscopy or other interventions were required without the need for a follow-up appointment. The radiologist's report could assist hospital specialists in prioritising outpatient appointments [17]. Finally the estimated cost of accidents is £15 billion to the nation and approximately £1.2 billion per annum to the National Health Service (NHS) [18]. Early access to MRI through referral from primary care could contribute to the diagnosis and management of these patients and potentially prevent the onset of chronic problems and the psychological and economic consequences of loss of physical fitness.

In contrast, some take the view that patients benefit more by being referred quickly and directly by their GP to see an orthopaedic specialist [8]. This would allow hospital specialists to use MRI much more selectively, limiting it to those patients for whom a decision to operate has already been made and thus reduce resources spent on MRI. They argue that imaging may confuse the clinical picture if it detects asymptomatic abnormalities, possibly leading to unnecessary referrals and interventions [19]. Not all GPs understand the implications of MRI findings as reported by radiologists, and this could result in false reassurance and delays in appropriate treatment [20].

A survey of the availability of MRI discovered that of 121 public sector departments with MRI who responded, 74 (61%) provided direct access to GPs for imaging of the knee [21]. These findings imply wide variation in GPs' access to MRI and provide further evidence of the uncertainty where MRI should enter the diagnostic pathway. To the extent that the distribution of scanners reflects past demands of fundholding GPs, this variation may be more politically driven than evidence based. Furthermore referral behaviour varies among GPs with direct access to MRI. How such access affects the case mix of patients referred to orthopaedic clinics is not known. Investment in primary care is increasing, not least to prevent unnecessary referral to secondary care; Primary Care Trusts will control over 80% of the NHS budget by 2007/8 [22]. In addition, the UK Department of Health has announced plans to reduce waiting times for diagnostic tests [23]. MRI provision is expected to rise by around 12% a year [24]; nearly one million MRI examinations are now performed in England each year [25]. There is a real danger that GP access to MRI will become standard policy without rigorous evaluation. In contrast, timely access to a reliable diagnostic tool in primary care has potential for better care and reduced costs.

So our multi-centre, pragmatic randomised trial with two parallel groups and concomitant economic evaluation addresses the question whether patients presenting to GPs with suspected internal derangement of the knee should be referred for MRI or directly to an orthopaedic specialist? The principal objectives are to evaluate: whether the use of MRI in primary or secondary care affects subsequent diagnosis and management; whether it improves patient outcomes; and whether it reduces net costs to the NHS, patients and society. In summary, our trial will inform policy whether to allocate resources to permit MRI for suspected internal derangement of the knee to enter the diagnostic pathway in primary care through early access for GPs, or to restrict it to secondary care at the request of orthopaedic specialists.

## Methods/Design

### Trial design (Figure 1)

Experience from a wide range of trials in primary care suggests that obtaining consent within a single consultation is difficult. Therefore all potential trial participants were given an information leaflet by their GP at the first consultation. Patients who were then eligible, but uncertain about whether to participate were encouraged to take at least 24 hours to decide.

In each practice participating GPs or practice nurses randomised eligible and consenting participants to one of the two trial interventions: referral to the local radiology department for an MRI examination underpinned by a provisional orthopaedic referral; or referral as usual to the local orthopaedic department for consultation with a specialist.

### Interventions

#### Direct access to MRI (experimental intervention)

At each hospital, imaging was performed with standard commercially available MR imagers using imaging protocols at the discretion of the radiologist. 'Excess treatment costs' were used to ensure that participants in the experimental group could have early access to MRI within twelve weeks of referral from their GP, who then used the MRI findings to inform diagnosis and plan subsequent management. Participants with normal MRI findings received treatment including advice to return to normal activities, undertake quadriceps exercises, and referral for physiotherapy depending on their clinical history. GPs were advised that when radiologists reported a serious abnormality like a tumour on an MRI examination the participant should be fast-tracked as normal.

To avoid contaminating our evaluation by differences in waiting times between the two clinical policies, we asked GPs to make a provisional referral to orthopaedics at the same time as requesting MRI. This was to ensure that the total waiting time from GP consultation to orthopaedic appointment was similar for both trial arms. Thus the trial will establish the more appropriate sequence of events rather than try to assess the influence of variations in waiting times.

**Figure 2:**
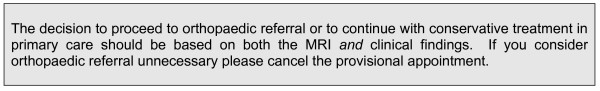
Educational reminder.

There is evidence that educational interventions to support the dissemination of clinical guidelines can improve GPs' knowledge of MRI use [26] and the routine attachment of educational messages to radiologists' reports can avoid unnecessary GP referral [27]. We therefore delivered educational seminars to GPs about MRI, clinical diagnosis and conservative management and attached the educational message shown in Figure 2 to the radiologists' reports.

#### Referral to orthopaedic specialist in secondary care (the control intervention)

The potential delay between GP referral and a participant's outpatient appointment with an orthopaedic specialist varied across experimental sites from three to eighteen months. We therefore used 'excess service support costs' when necessary to run extra clinics to reduce the waiting time for an orthopaedic appointment from GP referral to within nine months, and the delay between orthopaedic consultation and arthroscopy to within nine months. Reducing the waiting time from GP referral to arthroscopy to eighteen months meant that following up participants for two years would ensure the evaluation covered most relevant events including arthroscopy. Orthopaedic specialists decided whether to request MRI as normal.

### Inclusion and exclusion criteria

The target population for inclusion were people aged between 18 and 55 years inclusive presenting in general practice and for whom the GP was considering referral to see an orthopaedic specialist for suspected internal derangement of the knee (e.g. meniscal or ligamentous injuries). Individual practices decided whether GP Registrars should recruit and manage eligible patients. Consultant radiologists could delegate reporting to Specialist Registrars. Consultant orthopaedic surgeons could also delegate to junior staff if appropriate.

Patients were excluded if:

• Their GP judged that they needed urgent orthopaedic referral at the initial consultation (e.g. gross ligamentous injury or sudden onset of effusion).

• They had suspected osteoarthritis, other non-traumatic arthropathy, or isolated patello-femoral joint pain.

• They had chronic instability of the knee due to history of major injury.

• They had a previous MRI examination within the same episode of care.

• They had previous surgical intervention (excluding diagnostic arthroscopy) on the same knee.

• They had contraindications to the use of MRI, for example pacemaker, intra-cranial aneurysm clips, or orbital metallic foreign body.

**Figure 3:**
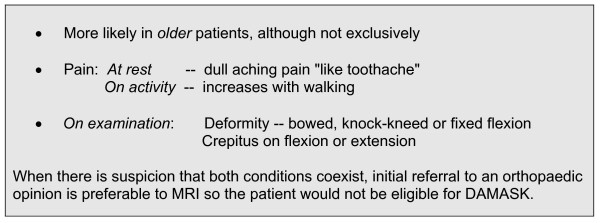
Distinctive features of osteoarthritis rather than internal derangement of the knee.

**Figure 4:**
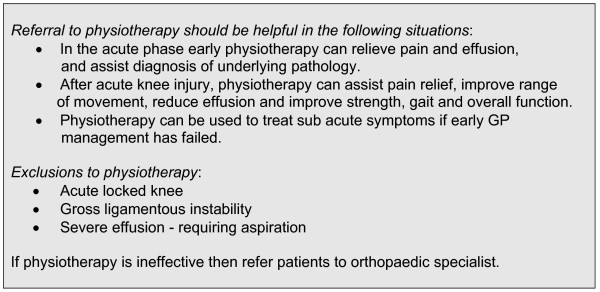
Reasons to refer for physiotherapy.

Figures 3 and 4 summarise the guidance given to GPs to differentiate between osteoarthritis and internal derangement of the knee and how physiotherapy might be useful before referral to an orthopaedic specialist.

### Recruitment and allocation to interventions

The trial was based in sites across North Wales, North East Scotland, and Yorkshire – areas covering urban, mixed and rural settings and a broad socio-economic spectrum. The total population of these geographical areas is around two million people registered in over six hundred general practices.

There is evidence that a multi-faceted strategy including education, financial compensation and regular contact is effective for recruiting GPs and patients [28]. Therefore when practices were approached to participate in the trial we offered GPs the opportunity to attend seminars to learn about and discuss the trial and the choice of receiving payment or postgraduate education accreditation. The seminars were held at places and times most convenient for GPs. We also visited the practices of GPs unable to attend the seminars and contacted others by telephone. To cover expenses each participating practice received payments for committing themselves to the trial, recruiting participants, and for completing all relevant documentation. During recruitment we updated participating practices through a newsletter, and maintained regular contact by telephone and email. Posters were designed to alert patients with knee problems about the study whilst waiting for their appointment in general practice. We publicised the trial through primary care newsletters, articles in local newspapers, the trial website, and leaflets and posters in local Accident & Emergency and Physiotherapy Departments, pharmacies and sports centres. We also asked hospitals to ensure that patient contact with MRI or Orthopaedic Departments triggered referring GPs from participating practices to consider recruiting a patient into the trial if they had not already done so.

#### Ethical approval

The trial protocol was designed to comply with the Declaration of Helsinki as adopted by the World Medical Association. Northern and Yorkshire Multi-Centre Research Ethics Committee approved the protocol (reference number MREC/1/3/59).

#### Obtaining consent

When patients consulted their GP with a knee problem, the GP provided the patient with the information leaflet, unless there was any obvious reason for exclusion. Practices that collected computerised data on consultations were also asked to identify and send leaflets to patients not previously identified at the time of their initial consultation who might still be eligible. Patients who were eligible and agreed to consent were asked by their GP to complete the baseline questionnaire.

#### Randomisation

When a participant completed the baseline questionnaire a designated member of the practice phoned the remote randomisation service at the University of York Trials Unit for the random allocation. They then informed the participant of their allocation and the GP made the appropriate referrals. As the trial was pragmatic in design, so as to reflect the consequences of routine GP access to MRI, blinding of participants or professionals to treatment allocation was not appropriate.

The randomisation service ensured immediate and unbiased allocation of individual participants between the two arms of the trial. The service recorded information to identify all participants and their eligibility. There is evidence of substantial inter-observer variability in radiologist reporting of MRI of the knee [29]; there is also evidence that practice list size [30] and distance from general practice to MRI centre [31] influence referral for MRI. We therefore stratified the randomisation procedure by experimental site, median distance from practice to hospital, and median number of partners in practices as a proxy for practice list size. Within strata a block allocation sequence was used; permuted random blocks of size 2 or 4 were randomly selected to generate the allocation sequence.

When the trial began we recruited participants from practices in Hull and East Yorkshire, York and North Yorkshire, Grampian and North Wales. Delays, for example in the approval of NHS costs from North Wales, led us to extend this invitation to practices to participate in Lothian, Bradford, Rotherham, Sheffield and Tayside. We modified the randomisation procedure for practices from these sites to reduce administration and expedite recruitment. The new procedure required the GP to establish eligibility, obtain consent, make the orthopaedic referral, and provide the participant with the baseline questionnaire and contact details form. The participant posted completed forms to York where the Trial Secretary entered the data into the randomisation database, performed the randomisation, and posted the results of the allocation to the GP and the participant.

#### Stopping rules

Participants referred to orthopaedic departments were at no greater risk than in normal clinical practice. MRI of the knee has no serious side-effects providing the known contraindications are avoided, which is routine practice in Radiology departments. A very small percentage of patients find lying in the scanner space unpleasant [32]. As this is thus a pragmatic trial with very little risk to participants, interim analyses were unnecessary.

### Outcome measures

Participants completed a baseline questionnaire after giving informed consent but before randomisation. The main outcome measures were self-assessed questionnaires asking about participants' knee-related health, general health, and demographic characteristics. Participants received similar questionnaires by post six, twelve, and 24 months after random allocation. Existing evidence about effective follow-up strategies enabled us to maximise the effective sample size and reduce bias [33]. At six months follow-up this included a reminder by post after two and four weeks and by telephone after six weeks if necessary. At twelve and 24 months this strategy was supplemented with a two week pre-notification letter enclosing £5. This money was given whether or not participants completed questionnaires to cover expenses incurred in doing so. At the final reminder six weeks after the 24 month follow up, participants who had not returned the questionnaire could choose to complete an abridged questionnaire by telephone. This comprised the EQ-5D and five questions from the knee-specific instrument that explained most of the variation in participants' responses.

#### Health outcomes

In the absence of an appropriate patient-assessed health instrument specific to the knee with satisfactory evidence for reliability, validity, and responsiveness [34], we developed our own instrument. We complemented this instrument with two generic measures useful for identifying unexpected effects of interventions and for economic evaluation. The Short Form 36-item (SF-36) health survey is a popular health profile that has been validated for use in the NHS [35]. The EQ-5D generates a single index for valuing health states and is therefore suitable for cost-utility analysis [36]. Both measures are responsive to changes in the health of patients referred for MRI of the knee [37].

#### Health economic outcomes

Musculoskeletal problems and associated disabilities generate high costs – in health care (both within and outwith the NHS), social security, and lost production [38]. A broad economic evaluation is essential to inform commissioning and service decisions about the most efficient policy for managing patients with continuing knee problems.

To estimate the incremental costs of each policy the economic evaluation takes the perspective of both the NHS and society. When we have completed the prospective collection of data from participants, practices and hospitals on NHS resources consumed we shall use these data to estimate both the quantity of resources consumed and complement this with the unit cost of each resource. Unit cost data will be derived from reliable published sources [39], the Department of Health Central cost estimates, and from manufacturers.

Thus the data available for economic analysis are patient-specific resource use and costs. Given the skewness inherent in most cost data and the focus of analysis on mean costs, we shall use bootstrapping to estimate confidence intervals around the difference in mean costs [40,41]. The base-case analysis will consider the costs and consequences of direct access to MRI, reporting disaggregated data on incremental costs and on the broad range of consequences. The base-case analysis will consider both the NHS perspective and a broader societal view where patient costs and productivity issues will additionally be incorporated. If one intervention clearly dominates the other in both costs and consequences, then analysis will be essentially complete. If there is no such dominance, however, we shall use both cost-effectiveness (focusing on cost per change in knee specific score) and cost-utility analyses (focusing on cost per quality-adjusted life year (QALY) gained), as estimated from the EQ-5D instrument. Results will be presented using cost-effectiveness acceptability curves to reflect sampling variation and uncertainty in the threshold value of a QALY. We shall also use simple and probabilistic sensitivity analyses to explore whether these results are robust to plausible variations in key assumptions (e.g. average appointment times) and variations in analytical methods, and to consider the generalisability of the results.

#### Referral process

To measure 'diagnostic and therapeutic impact' of direct access to MRI, we asked GPs to complete a pre-randomisation questionnaire that recorded their diagnosis, management plans and confidence therein [42]. We asked them to complete a similar questionnaire on receipt of the imaging report or orthopaedic specialist letter. We also record the patient waiting times from randomisation to MRI, orthopaedic consultation, and arthroscopy. Adherence to the referral process is one of the outcomes of interest rather than a factor that may jeopardise interpretation of the findings.

### Sample size

A similar group of patients followed up six months after referral for MRI of the knee had a mean of 64 and standard deviation (SD) of 25 on the physical functioning sub-scale of the SF-36 [37]. Hence a trial that followed up 434 participants (217 direct access and 217 controls) would have 80% power using a 5% significance level to identify a standardised difference of 0.27, equivalent to 6.75 points on the SF-36 physical sub-scale or an analogous difference on the proposed knee-specific instrument. We judge that a standardised difference of 0.27 in either of these measures should be clinically important. As we estimated that we could achieve 85% response rates to postal questionnaires, we aimed to recruit 500 participants in all.

### Statistical analyses

The primary outcome measures are the physical functioning sub-scale of the SF-36 and the knee-specific instrument. The trial is pragmatic in design and therefore the primary analysis will be 'by intention to treat' in that all participants properly randomised will be included in the analysis even if they do not receive the intervention they were allocated to receive. A secondary analysis will be limited to participants who received the treatment to which they were randomised.

Data were collected at four time points: baseline, six, twelve, and 24 months. We shall use PROC, the mixed model procedure in the Statistical Analysis System (SAS), to analyse the data from all four time points within a single model and to adjust for experimental site. This model is robust to data missing at random and can also take the covariance structure of the data into account [43]. We shall check that the data fulfil the assumptions of the mixed model. If the data are not missing at random we shall seek an alternative analytical method providing a better fit. We shall treat missing items within individual outcome measures according to the instructions for that measure. For each outcome measure we shall calculate the number of non-responders and compare the proportion and type of non-response in each group at each time point. We shall also check that the model is a good fit and transform the data if necessary to improve fit.

The secondary outcome measures are the other seven subscales of the SF-36, the EQ-5D itself, the number of days patients take off work for their knee problems, and the number of days they are prevented from doing normal activities by their knee problems. The EQ-5D and the other seven subscales of the SF-36 will be analysed the same way as the primary outcome measures. As the number of days off work or normal activities is likely to follow a skewed distribution we shall transform the data and present the median and inter-quartile ranges for these times.

Secondary analysis of the primary outcome measures will be performed to adjust for the variation in waiting times between GP referral and access to MRI or orthopaedic specialist. Further analyses will test whether variables such as age and sex affect patient outcome, and to compare findings between different types of knee injuries (e.g. meniscal or ligamentous). As this analysis will not have the same power as the trial as a whole it will generate hypothesis rather than test them definitively.

## Discussion

We have designed the DAMASK trial to evaluate the role of MRI within primary care and contribute to improved communication and evidence-based partnerships between primary and secondary care professionals.

## Competing interests

The author(s) declare that they have no competing interests.

## Authors' contributions

SDB, SB, FJG, HH, DK, ITR, MR and CW were responsible for the research question and conception of the study. SDB, SB, MR, CA, SC, HC, BC, FF, AG, MGG, MH, KH, VM, JO, DT and VW participated in the implementation of the study protocol. SDB, MGG and MH co-ordinated the whole trial. SB was responsible for writing this manuscript. All authors read, commented on, and approved the final manuscript.

## Pre-publication history

The pre-publication history for this paper can be accessed here:



## Figures and Tables

**Figure 1:**
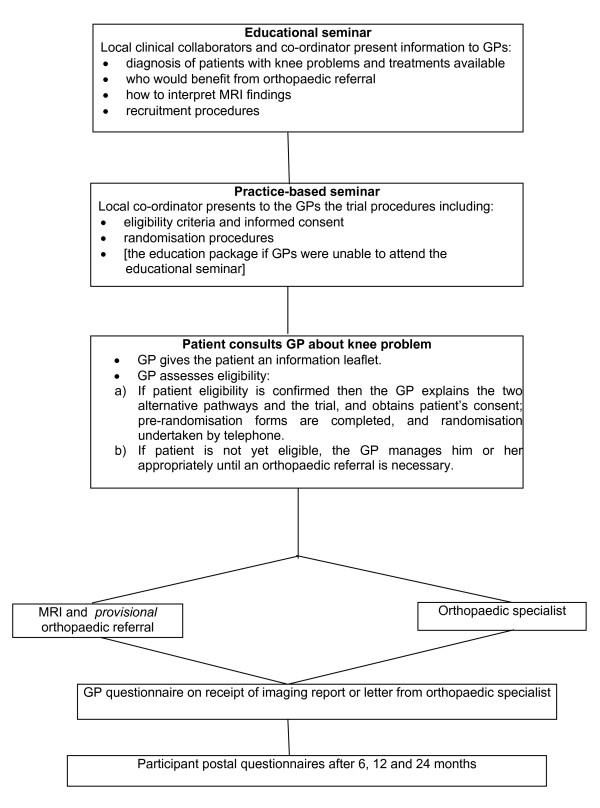
Trial Design.
